# The Therapeutic Potential of the Ketogenic Diet in Treating Progressive Multiple Sclerosis

**DOI:** 10.1155/2015/681289

**Published:** 2015-12-29

**Authors:** Mithu Storoni, Gordon T. Plant

**Affiliations:** Department of Neuro-Ophthalmology, The National Hospital for Neurology and Neurosurgery, Box 93, Queen Square, London WC1N 3BG, UK

## Abstract

Until recently, multiple sclerosis has been viewed as an entirely inflammatory disease without acknowledgment of the significant neurodegenerative component responsible for disease progression and disability. This perspective is being challenged by observations of a dissociation between inflammation and neurodegeneration where the neurodegenerative component may play a more significant role in disease progression. In this review, we explore the relationship between mitochondrial dysfunction and neurodegeneration in multiple sclerosis. We review evidence that the ketogenic diet can improve mitochondrial function and discuss the potential of the ketogenic diet in treating progressive multiple sclerosis for which no treatment currently exists.

## 1. Introduction

Jean-Martin Charcot's careful clinical and pathological account of a patient with demyelinating lesions or “sclérose en plaques” in 1868 provided the world with the first clear description of multiple sclerosis (MS) [1]. Despite almost a century and a half of our acquaintance with this disease, its pathogenesis remains elusive and we continue to lack therapeutic options to treat progressive MS [2].

Eighty-five per cent of cases of MS present with recurring episodes of isolated neurological syndromes, which usually resolve with conservative management (termed relapsing and remitting or RRMS). The remaining 15% of cases present with a gradual and progressive loss of neurological function that does not recover (termed primary progressive or PPMS). Of the 85% of the cases who present with RRMS, the majority begins to suffer from progressive neurological decline after one to three decades (termed secondary progressive or SPMS) that is similar in pattern to PPMS [3, 4].

MS has traditionally been viewed as an immune-mediated inflammatory disease. An immune response is thought to be responsible for causing the spontaneously remitting relapses in RRMS. Immune cells migrate across a compromised blood brain barrier and cause the focal and disseminated inflammation typical for RRMS. The traditional view of MS as an inflammatory disease has resulted in almost all therapeutic strategies taking an immunomodulatory or immunosuppressive approach [2].

The idea of MS having a primarily inflammatory component is challenged by the observation that focal inflammation may be absent in a minority of cases of progressive MS [5]. Neurodegeneration may play a more central role in its pathogenesis [6]. As the majority of current therapeutic options for MS are based on immune therapy, there is at present no definitive therapy for progressive MS, whether primary or secondary [7].

Accumulating evidence of a dissociation between inflammation and disease progression warrants a revised perspective on the role of neurodegeneration in the pathogenesis of MS and therefore the therapeutic strategies. In this review, we explore the evidence of neurodegeneration and the players in its pathogenesis that lead us to the potential of the ketogenic diet for use as a therapy for progressive MS.

## 2. Is MS Primarily a Neurodegenerative Disease?

The traditional model of MS is based on an “outside-in” approach. In this model, a dysregulated immune system attacks the central nervous system. Peripheral immune cells cross a compromised blood brain barrier to enter the central nervous system and give rise to acute multifocal inflammatory lesions, which are either asymptomatic or manifest as relapses in RRMS involving a variety of neurological symptoms disseminated in time. Although RRMS is the commonest mode of MS presentation, the vast majority of patients presenting in this way enter into a progressive phase of MS (SPMS) as long as three decades after disease onset.

The duality of the inflammatory and neurodegenerative components is made salient by the observation that MS can “worsen” at exactly the same rate regardless of whether a patient started off with decades of isolated inflammatory relapses or entered directly into progressive MS. Pathophysiologically, no difference can be found between the two disease phenotypes [8, 9].

### 2.1. An Alternative Model of MS

Such evidence of a distinct dissociation between disease progression and inflammation has challenged the traditional “outside-in” approach. Progression in the absence of immune attacks is indicative of a separate and parallel pathogenic process. Some authors have proposed an “inside-out” model of MS where primary cellular degeneration is the initiating factor that then triggers inflammation [10]. The degeneration releases antigenic cellular matter that then invites an immune response.

### 2.2. Halting Inflammation Does Not Halt Disease Progression

Although there is as yet no conclusive evidence to suggest that degeneration might be the initial event that triggers the inflammation seen in MS (the age-old chicken and egg question), clinical findings support a dissociation between the two, where, at the very least, degeneration does not follow inflammation and may occur independently of inflammation. Most varieties of immune-modulating therapy that reduce and even eliminate inflammation have little bearing on the progress of MS in the very long-term, although a few of the newer immunomodulatory agents may hold greater promise [11–14]. Autologous haematopoietic stem cell transplantation therapy, though highly efficacious at reducing inflammation, does not halt axonal degeneration and brain atrophy [15]. Progressive neurodegeneration and axonal atrophy in the absence of inflammation are observed in MS [16].

### 2.3. Harding Syndrome

There is pathological evidence in support of the theory that neurodegeneration may occur in complete isolation in MS without any evidence of preceding inflammation: foci of damage have been observed in the inner layers of the myelin sheath with the outer layers remaining intact. This starkly challenges the possibility of a T cell mediated external mechanism [17, 18]. In Harding syndrome, there is evidence that neurodegeneration precedes and then causes the neuroinflammation seen in classical MS. In this condition, the cellular degeneration occurring as a result of defective mitochondrial function may trigger an autoimmune response in those who are “immunologically primed” [19]. Although Leber Hereditary Optic Neuropathy (LHON) is more penetrant in males, females are at greater risk of autoimmune disease and this may explain why there is a higher incidence of Harding syndrome with MS-like inflammation in females, despite involving the same mutation as LHON [20].

## 3. The Role of Mitochondria in Multiple Sclerosis

Whether neurodegeneration triggers inflammation or is triggered by inflammation or occurs as a parallel process, the evidence that MS involves neurodegeneration as well as neuroinflammation is ever increasing. Mitochondrial dysfunction is thought to play a central role in the neurodegenerative disease process and a growing body of evidence suggests that mitochondrial dysfunction may also be of great importance in the pathogenesis of MS [21, 22].

### 3.1. Mitochondrial Function May Determine the Fate of “Struggling” Axons

Axonal degeneration is a prominent feature of MS and is notably present even in the absence of local demyelination [23, 24]. Animal models suggest that mitochondrial injury may be a necessary step preceding axonal degeneration. The generation of reactive oxygen species may contribute to mitochondrial injury as the detoxification of reactive oxygen species reverses the injury and halts axonal degeneration [25].

Once chronically demyelinated, some axons degenerate while others may survive. It seems possible that mitochondrial “stealth” may determine the fate of demyelinated axons. Degenerating axons are seen to contain dysfunctional mitochondria, whereas the axons that survive demyelination contain highly functional mitochondria with increased respiratory chain activity [26, 27]. These findings mirror a recent study on glaucomatous optic neuropathy where it was observed that robust mitochondria may offer protection from neurodegeneration despite the presence of high intraocular pressures [28]. It is thought that, where axons degenerate following demyelination, they do so when energy production by mitochondria becomes inadequate. Mitochondrial function seems to determine the fate of axons under duress.

### 3.2. Mitochondrial Dysfunction Is Seen in “Normal Appearing” Grey Matter

Grey matter atrophy is a recognized feature of MS and the rate of atrophy is seen to increase as the RRMS stage progresses to SPMS [29]. Furthermore, neuronal atrophy can be seen to occur independently of demyelination, in areas of “normal appearing grey matter” as ascertained by immunohistochemical staining techniques and microscopy [30]. Mitochondrial function within cortical neurons in MS has been shown to be compromised. Campbell et al. (2011) used complex IV/complex II histochemistry, immunohistochemistry, laser dissection microscopy, and PCR and DNA sequencing methods to demonstrate a striking reduction in the activity of complexes II and IV within the oxidative phosphorylation chain in neurons obtained from autopsies of cases of SPMS [31].

### 3.3. Progressive Mitochondrial Compromise May Correlate with Reduced Recovery from Relapses

Levels of the transcriptional cofactor PGC-1a, which plays a key role in the activation of nuclear transcription factors involved in mitochondrial function, may be reduced in cortical neurons in progressive MS. Its expression was found to correlate with neuronal density [32]. Given the observation that the rate of brain atrophy increases as RRMS progresses to the SPMS, the decline in PGC-1a may suggest a parallel worsening in mitochondrial function [33]. During this stage of progression, recovery from relapses becomes progressively incomplete [34]. The progressive decline in mitochondrial function with a resulting decrease in ATP availability may plausibly cause a decline in axonal resilience, making recovery from each episode of relapse increasingly difficult.

A reduction in PGC-1a levels has also been observed in other neurodegenerative conditions such as Alzheimer disease [35].

## 4. Mitochondria and Neurodegeneration

Several groups have proposed a model for neurodegeneration in which mitochondrial dysfunction is central to its pathogenesis [36, 37]. In this model, mitochondrial dysfunction precedes synaptic dysfunction, atrophy, and neuronal loss.

In an animal model of MS, experimental autoimmune encephalomyelitis (EAE), mitochondrial injury has been shown to precede inflammation and to be the trigger for neurodegeneration [38]. Although the precise molecular pathways leading to mitochondrial damage remain unknown, oxidative damage is one possible route [39].

Early studies of antioxidant therapies in animal models of MS are showing promising results. Superoxide dismutase 2 has been shown to salvage axonal loss in EAE-related optic neuritis [40]. A synthetic antioxidant, Mito-Q, has been shown to be neuroprotective and delay disease progression in EAE, despite having no effect on inflammation, further confirming a dissociation between the two disease processes and demonstrating that neurodegeneration might be more worthy of therapeutic intervention in order to improve disease prognosis [41].

To date, one of the few available therapeutic options used in MS that may have some beneficial effects on progressive MS is Dimethyl Fumarate or DMF [42]. DMF is the only current therapy for MS that in addition to having an immunomodulatory effect is also a potent antioxidant agent. It is thought to reduce oxidative stress through the NRF-2 pathway and hence have a neuroprotective effect [43]. This neuroprotective effect has also been evident in other neurodegenerative settings [44].

## 5. Mitochondria as the Therapeutic Target for Progressive MS

The paucity of treatment options for progressive MS indicates an urgent need to find therapeutic targets. The role of mitochondrial dysfunction in neurodegeneration suggests that targeting mitochondrial function may be a useful therapeutic strategy for progressive MS although clinical trials examining the efficacy of agents promoting mitochondrial function in non-MS diseases with known mitochondrial pathology have shown variable results. Some of this variability may stem from differences and difficulties faced with drug delivery and dosage [45].

There are a limited number of studies to date testing the efficacy of agents that target mitochondrial function in the setting of MS, but they offer considerable promise in the efficacy of mitochondria-targeted therapy. Coenzyme Q10 has antioxidant properties and forms part of the electron transport chain interacting with complex I. A randomized placebo-controlled double-blind study of Coenzyme Q10 supplementation over 12 weeks in patients with relapsing and remitting MS demonstrated a reduction in IL-6 and MMP-9 levels [46]. The results of another similar trial by the same group demonstrated a reduction in depression and fatigue [47].

Mouse models of MS (EAE) are based on an immune/inflammatory model and not a degenerative model limiting the ability of the mouse model to accurately reflect the oxidative damage occurring in human MS. This may explain some inconsistencies in the results obtained from mouse model studies of MS [48, 49]. Mito-Q, an antioxidant that contains ubiquinone, has been shown to delay disease progression and reduce neuronal cell loss in a mouse model of multiple sclerosis; however, one study using a synthetic analogue of Coenzyme Q10 failed to prevent disease progression [50].

## 6. Glucose Hypometabolism

Some studies have suggested that there may be a bioenergetic shift taking place within neuronal metabolism prior to the onset of clinical signs of neurodegeneration, where glucose uptake and utilization become progressively reduced. This glucose hypometabolism may reflect a decline in mitochondrial function. The shift has been observed to occur long before the onset of clinical signs of neurodegeneration, suggesting the possibility that glucose hypometabolism may be the initial step leading to axonal atrophy and neuronal loss through a reduction in ATP availability [51–54]. The bioenergetic shift appears to specifically affect the metabolism of glucose. No such shift is seen with ketone body metabolism [54, 55].

### 6.1. Glucose Hypometabolism in MS

The neurodegenerative process underlying progressive MS may also result in glucose hypometabolism. This would suggest a potential therapeutic advantage in boosting energy supply through an alternative route, such as ketone metabolism. A study that compared 47 MS patients with varying levels of fatigue and 16 healthy controls showed that the patients had reduced cerebral glucose metabolism in various regions within the brain, including the prefrontal, premotor, and supplementary motor areas and the putamen when compared to control subjects. There was an inverse correlation between degree of fatigue and glucose metabolic rate [56]. Another study on 8 MS patients and 8 gender matched healthy control subjects demonstrated lower glucose uptake in 40% of the brain compared to healthy controls [57].

Extramitochondrial metabolism increases in the presence of impaired mitochondrial metabolism of glucose. In a pilot study comparing 85 patients with relapsing and remitting MS and 54 patients with secondary progressive MS as well as 18 healthy controls, extramitochondrial glucose metabolism showed a correlation with disease progression, suggesting that impaired mitochondrial metabolism of glucose may play a significant role in disease progression in progressive MS [58].

Further molecular evidence of impaired glucose metabolism playing a role in MS is seen in the altered distribution of glucose (GLUT) and monocarboxylate transporters (MCT) within chronic lesions of MS where there is a decline in axonal GLUT3 and MCT2 expression. These changes may confer resistance to glucose entry into demyelinated axons, depriving them of adequate fuel supply resulting in glucose hypometabolism [59].

The possibility that providing the brain with an alternative source of fuel may reduce the rate of neurodegeneration is a promising avenue to explore, particularly where there remains a paucity of therapeutic options [60].

## 7. The Potential of Ketones to Provide an Alternative Fuel Supply

In 1967, Cahill et al. demonstrated that, during prolonged fasting, the body provides the brain with an alternative source of fuel, in the form of ketone bodies. The central nervous system is unable to use fat as a direct energy source and after prolonged carbohydrate restriction, fat is converted into ketone bodies in a process referred to as “ketogenesis.” Ketogenesis takes place primarily within the matrix of mitochondria located within the liver. Ketogenesis results in the production of the ketone bodies beta-hydroxybutyrate, acetoacetate, and acetone which replace glucose as the brain's main sources of fuel [61]. Hans Krebs first made the distinction between the normal, “physiological” ketosis that is induced when following a carbohydrate-restricted diet where levels of ketones do not exceed 8 mmol/L and diabetic ketoacidosis, a complication of diabetes where ketonaemia can exceed 20 mmol/L and result in acidosis [62]. Ketone bodies can easily cross the blood brain barrier and the brain's usage of ketone bodies increases as their concentration in the serum increases, up to a concentration of 12 mmol/L [63]. A meta-analysis of animal studies has shown that the cerebral metabolic rate of glucose decreases 9% with every 1 mmol/L increase in total plasma ketones. Ketones bypass the glycolytic pathway and directly enter the Tricarboxylic Acid (TCA) cycle within mitochondria, contributing to anaplerosis.

## 8. A Ketogenic Diet for the Neurodegenerative Component of Progressive MS

The ketogenic diet has traditionally been used for the treatment of resistant epilepsy, but it is increasingly becoming apparent that its benefits may apply to a wider spectrum of neurological disease. Although research on its use outside the realms of epilepsy is still at its infancy, the findings are promising and hold great potential for the treatment of neurodegeneration, particularly with regard to mitochondrial function.

A ketogenic diet has a favourable effect on mitochondrial function. It reduces levels of reactive oxygen species and increases ATP availability. The diet may provide neuroprotection and reduce inflammation. Ketones produced during a ketogenic diet can be used as an alternative source of fuel in the setting of impaired glucose metabolism.

## 9. The Effect of the Ketogenic Diet on Oxidative Stress

A ketogenic diet has been shown to reduce the generation of reactive oxygen species through its effect on uncoupling proteins. It also increases levels of antioxidant agents including catalase and glutathione through its inhibitory action on histone deacetylases and activation of the Nrf2 pathway.

### 9.1. The Ketogenic Diet Increases Mitochondrial Uncoupling Protein Levels

The process of oxidative phosphorylation generates reactive oxygen species. The extent of reactive oxygen species generation correlates strongly with the potential difference across the inner mitochondrial membrane. Uncoupling proteins (UCPs) can reduce this potential difference by allowing the entry of protons into the mitochondrial matrix. Although this “mild” uncoupling may incur a small reduction in ATP generated through oxidative phosphorylation, its overall net effect is to enhance respiration and ATP levels through a reduction in reactive oxygen species formation and protection from apoptotic events [64]. A ketogenic diet appears to promote UCP activity, specifically the activity of UCP2, UCP4, and UCP5 with a corresponding decline in reactive oxygen species [65].

### 9.2. Ketones Inhibit Histone Deacetylases

The ketone beta-hydroxybutyrate has a direct, dose-dependent inhibitory activity on class I histone deacetylases (HDACs) including HDAC1, HDAC3, and HDAC4. The ketone acetoacetate has also been shown to inhibit class I and class IIa HDACs. Beta-hydroxybutyrate's inhibition of HDAC promotes the acetylation of histone H3 lysine 9 and histone H3 lysine 14 and increases the transcription of genes regulated by FOXO3A. These include genes leading to the expression of the antioxidant enzymes mitochondrial superoxide dismutase and catalase [66].

### 9.3. A Ketogenic Diet Leads to the Activation of the Nrf2 Pathway

The ketogenic diet raises glutathione levels in the hippocampus of rats [67]. This is thought to occur through the Nrf2 (nuclear factor erythroid 2-related factor) pathway. When the ketogenic diet is first initiated, there is a temporary increase in oxidative stress. This may be activating Nrf2, since, a week after the temporary rise in oxidative stress, there is increased expression of Nrf2. Three weeks after the start of the diet, oxidative stress declines to below baseline levels and Nrf2 remains raised [68].

## 10. The Effect of the Ketogenic Diet on ATP Levels

A ketogenic diet enhances ATP production. The administration of beta-hydroxybutyrate immediately following bilateral common carotid artery ligation in a mouse model of global cerebral ischaemia preserves ATP levels [69]. Feeding mice a ketogenic diet for three weeks resulted in increased levels of ATP and the ATP/ADP ratio in the brain [70].

The improvement in ATP levels may partly be explained through the ability of the ketogenic diet to reduce oxidative stress. Although the diet may reduce reactive oxygen species generation through an increase in UCP activity, any reduction in oxidative phosphorylation incurred through UCP activity is outweighed by the enhancement of respiration and associated ATP production occurring as a result of reduced oxidative stress.

A ketogenic diet also appears to preserve ATP levels in the event of mitochondrial respiratory chain dysfunction, possibly through the replenishment of TCA cycle intermediates [71]. Beta-hydroxybutyrate attenuates the decrease in ATP production caused by a defect in complex I of the electron transport chain. It is thought to increase levels of the TCA intermediate succinate, which bypasses complex I when entering the TCA cycle [65, 72]. This carries considerable implications for MS, since defects in complex I within the electron transport chain have been observed in white matter lesions as well as in “normal” regions of the motor cortex [39, 73]. Ketones can also preserve ATP levels if complex II of the electron transport chain is inhibited, but this effect shows some regional specificity [74].

## 11. The Effect of the Ketogenic Diet on Mitochondrial Biogenesis

Mitochondrial biogenesis within the rat hippocampus and cerebellar vermis is increased by the ketogenic diet [75, 76]. Although the precise pathway for this is not known, it is thought to involve the PGC1*α* family of transcriptional coactivators, which promote transcription factors including NRF-1, NRF-2, and ERR*α* [77].

## 12. The Effect of the Ketogenic Diet on Inflammation

The anti-inflammatory effect of a ketogenic diet has been demonstrated in a murine model of lipopolysaccharide-induced fever [78]. In a rat model of MS, the diet suppressed the expression of inflammatory cytokines and enhanced CA1 hippocampal synaptic plasticity and long-term potentiation, which resulted in improved learning, memory, and motor ability [79]. The anti-inflammatory effect of a ketogenic diet may partly be explained through the inhibition of the NLRP3 inflammasome by beta-hydroxybutyrate in a manner that is independent of starvation-induced mechanisms such as AMPK, autophagy, or glycolytic inhibition. The NLRP3 inflammasome is responsible for the cleavage of procaspase-1 into caspase-1 and the activation of the cytokines IL-1*β* and IL-18. Its inhibition prevents IL-1*β* and IL-18 generation and their downstream effects [80].

## 13. The Neuroprotective Properties of the Ketogenic Diet

Ketone bodies play a neuroprotective role in animal models of neurodegeneration [69, 81]. ATP-sensitive potassium channels (K ATP channels) located on the cell surface of neurons stabilize neuronal excitability. Ketones promote an “open state” of these channels and confer neuronal stability [82]. K ATP channels also play a role in mitochondrial function and in cell death. The “open state” of K ATP channels located on the inner mitochondrial membrane prevents the formation of mitochondrial permeability transition pores (MPTPs) that can lead to mitochondrial swelling and cell death. Acetoacetate and beta-hydroxybutyrate have been shown to increase the threshold for calcium-induced MPTP formation [83].

## 14. The Regional Variation of the Effect of Ketones in the Mouse Cerebellum

Despite these seemingly positive effects on mitochondrial bioenergetics, the effects of a ketogenic diet on mitochondria within the mouse brain are not homogenous and some results appear conflicting. Although the study on the murine model of EAE demonstrated improved CA1 synaptic plasticity, in another study, on rats, although a KD prevented age-related morphological changes within the outer layer of the dentate gyrus of the cerebellum, it produced negative changes within the CA1 region [84]. In a study on rats fed a ketogenic diet for 8 weeks, antioxidant status was elevated within the hippocampus but not in the cerebral cortex and antioxidant activity was seen to be reduced within the cerebellum [85].

## 15. Human Studies

At present, there is a paucity of human studies on the use of ketones/the ketogenic diet in neurodegenerative disorders. There exists one recent, randomized, double-blind, placebo-controlled study looking at the effects of ketones on a neurodegenerative phenotype in 152 participants with mild to moderate Alzheimer disease, which found an improvement in cognitive scores when the oral ketogenic compound AC-1202 was used over a 90-day period. This improvement was greater in patients lacking the APOE4 polymorphism [86]. Smaller studies in other neurodegenerative settings have provided similarly positive results [87].

There is evidence of glucose transporter dysfunction within axons subjected to chronic lesions in MS and studies on subjects with inherited glucose transporter dysfunction who followed the ketogenic diet have yielded positive results [59, 88, 89].

## 16. Conclusion

The ketogenic diet has the potential to treat the neurodegenerative component of progressive MS on the basis of the following observations obtained from in vitro and in vivo studies,

1. Neurodegeneration is thought to underlie the pathogenesis of progressive MS.
2. Mitochondrial dysfunction may result in reduced ATP availability. This may promote axonal atrophy, leading to degeneration. There is evidence of mitochondrial dysfunction within “normal appearing” grey matter and mitochondrial function appears to correlate with axonal survival.
3. According to in vitro and animal studies, the ketogenic diet increases ATP production, promotes mitochondrial biogenesis, and bypasses dysfunctional steps within the mitochondrial bioenergetic process, increases antioxidant levels and reduces oxidative damage. Since an increase in ATP and overall improvement in mitochondrial functioning correlates with axonal survival, the ketogenic diet may offer a therapeutic benefit for the neurodegenerative component of MS.

These premises are largely theoretical with regard to the contextual application of the ketogenic diet in MS as there is no data currently available on the ketogenic diet from human studies on MS. Animal models of EAE do not accurately represent the underlying pathogenesis of MS, since neurodegeneration does not play a significant role in EAE. Mitochondria-targeting agents, ketones, and the ketogenic diet have however shown positive results in several models of neurodegeneration, and given the complete absence of available treatment for progressive MS, the relatively safe option of a ketogenic diet deserves further investigation in the context of progressive MS.

Despite its high fat component, the ketogenic diet is safe and even beneficial for cardiometabolic risk factors [90]. It has been in continuous use for almost a century for the treatment of epilepsy and has shown good tolerability, even in children [91]. Current ketogenic diet protocols involve a range of options, which encourages patient compliance. Where compliance may pose a challenge, mimicry of various components of the ketogenic pathway through the use of ketone analogues may offer a palatable therapeutic option [92]. Supplementation with ketones to induce ketosis has also shown an acceptable safety and tolerability profile [93].

Current treatment options in MS affect immune function and relapse rate with little effect on disease progression. They are sometimes accompanied with significant side effects including lymphopenia, multifocal leukoencephalopathy, and malignancy [7]. Consequently, it may be more favourable for some patients to pursue a relatively risk-free dietary approach that has the potential to reduce disease progression without affecting immune response. In conclusion, the ketogenic diet deserves further investigation for the purposes of treating progressive MS for which there currently exists no treatment.
